# Handling of spurious sequences affects the outcome of high-throughput 16S rRNA gene amplicon profiling

**DOI:** 10.1038/s43705-021-00033-z

**Published:** 2021-06-29

**Authors:** Sandra Reitmeier, Thomas C. A. Hitch, Nicole Treichel, Nikolaos Fikas, Bela Hausmann, Amanda E. Ramer-Tait, Klaus Neuhaus, David Berry, Dirk Haller, Ilias Lagkouvardos, Thomas Clavel

**Affiliations:** 1 ZIEL Institute for Food & Health, Technical University of Munich, Freising, Germany; 2 Chair of Nutrition and Immunology, Technical University of Munich, Freising, Germany; 3 Functional Microbiome Research Group, RWTH University Hospital, Aachen, Germany; 4 Institute of Marine Biology, Biotechnology and Aquaculture, Hellenic Centre for Marine Research, Heraklion, Greece; 5 Joint Microbiome Facility of the Medical University of Vienna and the University of Vienna, Vienna, Austria; 6 Department of Laboratory Medicine, Medical University of Vienna, Vienna, Austria; 7 Department of Food Science and Technology, University of Nebraska-Lincoln, Lincoln, NE USA; 8 Division of Microbial Ecology, Department of Microbiology and Ecosystem Science, Centre for Microbiology and Environmental Systems Science, University of Vienna, Vienna, Austria

**Keywords:** Next-generation sequencing, Microbiome, Microbial ecology

## Abstract

16S rRNA gene amplicon sequencing is a popular approach for studying microbiomes. However, some basic concepts have still not been investigated comprehensively. We studied the occurrence of spurious sequences using defined microbial communities based on data either from the literature or generated in three sequencing facilities and analyzed via both operational taxonomic units (OTUs) and amplicon sequence variants (ASVs) approaches. OTU clustering and singleton removal, a commonly used approach, delivered approximately 50% (mock communities) to 80% (gnotobiotic mice) spurious taxa. The fraction of spurious taxa was generally lower based on ASV analysis, but varied depending on the gene region targeted and the barcoding system used. A relative abundance of 0.25% was found as an effective threshold below which the analysis of spurious taxa can be prevented to a large extent in both OTU- and ASV-based analysis approaches. Using this cutoff improved the reproducibility of analysis, i.e., variation in richness estimates was reduced by 38% compared with singleton filtering using six human fecal samples across seven sequencing runs. *Beta*-diversity analysis of human fecal communities was markedly affected by both the filtering strategy and the type of phylogenetic distances used for comparison, highlighting the importance of carefully analyzing data before drawing conclusions on microbiome changes. In summary, handling of artifact sequences during bioinformatic processing of 16S rRNA gene amplicon data requires careful attention to avoid the generation of misleading findings. We propose the concept of effective richness to facilitate the comparison of *alpha*-diversity across studies.

## Introduction

Since the late 2000s, high-throughput sequencing of 16S rRNA gene amplicons has become the most popular method for rapid analysis of the diversity and composition of complex microbial communities [1]. Despite its popularity and usefulness, the method is prone to technical artifacts at various levels of the workflow, from sample processing to data analysis. For the latter, one common approach that has been used for decades [2] and is included in many freely available processing pipelines [3, 4] consists of building clusters of sequences representing single microbial entities, also known as operational taxonomic units (OTUs), at a defined level of sequence identity determined by the user (usually >97% used as proxy for species-level diversity) [5]. Other strategies, such as exact/amplicon sequence variant (ASV) analysis [6], are available, but do not replace the relevance of OTU-based approaches, as both can be applied in a synergistic manner and generate complementary readouts. Importantly, diversity measures derived from both ASV- and OTU-based datasets are strongly influenced by the choice of parameters during analysis. Lack of standardization has led to inconsistent results and confusion in the field, such as with estimates for the number of bacterial species in the human intestine ranging from a few hundred to several thousand [7, 8]. Reference studies based on low-error amplicon analysis protocols or shotgun metagenomics suggested the detection of 150–200 species in one individual sample, albeit based on sample size <200 [9, 10]. Despite the widespread use of 16S rRNA gene amplicon sequencing approaches, it is still unclear which thresholds of occurrence are most suitable to help eliminating falsely detected taxa, hereon referred to as spurious taxa. A widely used strategy to exclude spurious taxa is to remove so-called singletons, defined as those molecular species represented by only one sequence across all samples analyzed. However, this approach is extremely sensitive to several factors, such as the number of samples in the dataset and sequencing depth achieved, and its suitability for removing spurious taxa has not been rigorously evaluated.

In the present study, we assessed filtering thresholds suitable for excluding spurious taxa from high-throughput 16S rRNA gene amplicon datasets. We used mixtures of known bacteria both in vitro (mock communities) and from gnotobiotic mice to determine a consensus threshold. In addition, we studied the effect of different filtering approaches on final readouts using both literature datasets as well as in-house sequence data generated by three independent sequencing facilities. We also investigated the occurrence of spurious taxa across a range of ecosystems to identify their potential origin. Of note, our purpose is neither to set rules that should be blindly followed by all nor to refute data published in the literature. Filtering strategies necessarily depend on the specific aims of a given study and the type of samples analyzed. Rather, we aim to draw attention to the fact that inadequate filtering of spurious taxa can easily lead to false interpretations that rapidly spread throughout the scientific community and beyond, and that this problem may be largely mitigated by easily implementable analysis practices.

## Methods

### Datasets and samples

**Fig. 1:**
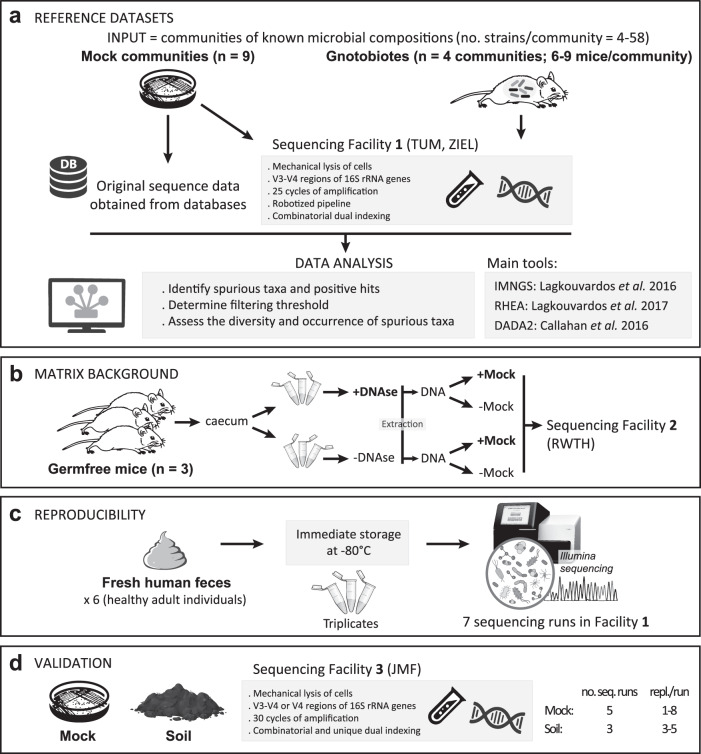
Schematic overview of the work. **a** The use of reference communities of microbes in vitro and in vivo using data from the literature or generated in-house and analyzed using different bioinformatic pipelines allowed precise analysis of the occurrence of spurious taxa. **b** Additional experiments using cecal contents from germfree mice in combination with DNase pre-treatment and mock DNA spiking were performed in a second sequencing lab to test effects of the matrix background on analysis outcomes. **c** Several human fecal samples stored under different conditions and processed in triplicates in different sequencing runs allowed assessing the reproducibility of microbiota profiles generated by high-throughput 16S rRNA gene amplicon sequencing following different filtering thresholds to remove spurious taxa. **d** Sequencing of a mock community and a soil sample, including several replicates and sequencing runs, followed by data analysis in a third facility were performed to validate findings. All technical details are given in the Methods section.

A schematic view of the main experiments included within the present study is provided in Fig. 1.

To determine filtering cutoffs, two types of reference communities were used: in vitro mixtures of known bacteria (mock communities) and in vivo communities from gnotobiotic mice, i.e., ex-germfree mice colonized with defined sets of known bacteria (Fig. 1a). Seven different mock communities from published studies with raw sequencing data available and two additional in-house generated datasets were used (Table 1). This work was complemented by the analysis of amplicon datasets generated from fecal samples of gnotobiotic mice colonized with four different mixtures of bacteria (Table 2).

**Table 1:** Mock communities used in the present study.

Name	Seq. facility^b^	Gene region	Replicates	No. species	No. raw reads	No. sequences after processing	Total no. taxa^d^ (no filtering)	1st spurious taxon^d^ (% rel. abundance)	Reference
Mock-1	See ref.	V4	1	27	153,841	140,397	432	0.007	[50]
Mock-2	See ref.	V4	1	58	593,868	578,569	761	0.439	[51]
Mock-3	See ref.	V4	1	21	1,012,097	453,215	1081	0.031	[52]
Mock-4	See ref.	V4	1	14	169,516	159,352	417	0.020	[53]
Mock-5	See ref.	V4	1	21	613,091	108,414	802	0.439	[52]
Mock-6	See ref.	V4	1	21	602,819	231,685	732	0.160	[52]
Mock-7	See ref.	V4	1	20	306,773	42,746	95	0.008	[40]
Mock-TUM	1	V3-V4	7	13	25,640 ± 8516	19,882 ± 8163	77 ± 15	0.130 ± 0.138	This study
ZymoBIOMICS (cat. #D6300)^a^	1	V3-V4	7	8^c^	67,465 ± 31,752	52,079 ± 24,759	177 ± 33	0.059 ± 0.026	This study
ZymoBIOMICS (cat. #D6311)^a^	2	V3-V4 and V4	25	8^c^	16,093 ± 9319	14,383 ± 9083	17 ± 15	0.106 ± 0.085	This study

In case of replicates, data are shown as mean ± SD.

The sequences of all species included in the respective mock communities are available via the project-specific data repository: 10.5281/zenodo.4837436.

^a^While D6300 corresponds to an evenly distributed mixture of the microbes, D6311 is a log-distributed mixture of DNA from the same microbes.

^b^For studies from the literature, please refer to the corresponding listed reference. For in-house generated data in this study: 1, ZIEL Core Facility Microbiome, TU Munich, Freising, Germany; 2, Joint Microbiome Facility of the Medical University of Vienna and the University of Vienna (JMF), Austria.

^c^Bacterial species only. The mock community includes also two yeast species not considered in the present study (ten microbial species in total).

^d^All values refer to OTUs clustered at 97% sequence identity, with exception of the last mock community analyzed in sequencing facility 2, for which values refer to ASVs.

**Table 2:** Gnotobiotic mouse communities used in the present study.

	Gene region	Replicates	No. species	No. raw reads	No. sequences after processing	Total no. OTUs (no filtering)	1st spurious OTU (% rel. abundance)	Reference
GNOTO1	V3-V4	6	7	28,706 ± 4904	25,869 ± 4494	66 ± 4	0.101 ± 0.014	This study
GNOTO2	V3-V4	9	12	30,261 ± 11,434	21,148 ± 8313	172 ± 24	0.009 ± 0.004	This study
GNOTO3	V3-V4	6	6	30,444 ± 42,325	27,632 ± 3563	85 ± 10	0.116 ± 0.016	This study
GNOTO4	V3-V4	7	4	25,217 ± 6514	47,505 ± 6106	68 ± 10	0.249 ± 0.041	This study

In case of replicates, data are shown as mean ± SD.

The sequences of all species included in the respective mock communities are available via the project-specific data repository: 10.5281/zenodo.4837436.

To analyze the impact of free DNA removal on data outcome (Fig. 1b), the cecal contents from three germfree mice were divided into two equal portions and processed with or without the iQ-Check Free DNA Removal Solution according to the manufacturer’s protocol (Bio-Rad Laboratories GmbH, cat. no. 3594970). After treatment, the solution was inactivated by heating (95 °C, 15 min). The DNA-extraction protocol described below in the section “Sample processing for sequencing at ZIEL and RWTH” was then used. Prior to library construction and sequencing at the RWTH Aachen, 20 ng of the ZymoBIOMICS DNA Standard (Zymo Research Europe GmbH, cat. no. D6306; 8 bacterial target species) was added to 1 µL of the DNA extract from germfree caecal content whenever appropriate. Besides the usual sequencing blanks described below, additional negative controls included the DNA samples from germfree caeca (both with and without DNA treatment) without addition of the ZymoBIOMICS DNA Standard. The latter was also sequenced as such to obtain a reference profile, including five different sequence cluster densities to assess the impact of sequencing depth on the occurrence of spurious taxa.

To further analyze the impact of different filtering strategies on data processing outcomes, two comprehensive studies with open access to their sequence data [11, 12] and data from six human fecal samples generated in multiple sequencing runs at the Core Facility Microbiome of the ZIEL Institute for Food & Health (Freising, Germany) were used (Fig. 1c). In order to validate findings in a third sequencing facility, amplicon datasets from one mock community and a peat soil sample were generated at the Joint Microbiome Facility of the Medical University of Vienna and the University of Vienna (JMF) (Austria), including multiple replicates and sequencing runs (Fig. 1d).

### Sample processing for sequencing at ZIEL and RWTH

DNA extraction and library preparation of mock communities and samples generated in the present study were performed as described previously [13]. Briefly, DNA was purified on columns (Macherey-Nagel; NucleoSpin gDNA Clean-up, cat. no. 740230.250) after mechanical lysis (bead-beating) and the V3-V4 region of 16S rRNA genes was amplified in a two-step approach (ZIEL, 15 + 10 cycles; RWTH, 15 + 15 cycles) [14] using primers 341F and 785R [15] following a combinatorial dual (CD) indexing strategy. Libraries were purified using magnetic beads (Beckman Coulter), pooled in equimolar amounts and then sequenced in paired-end mode (2 × 275 nt) using the v3 chemistry on an Illumina MiSeq following the manufacturer’s instructions. The platforms were semi-automated (Biomek4000 pipetting robot, Beckman Coulter) to increase reproducibility. Moreover, the workflow systematically included two negative controls (a DNA-extraction control, i.e., sample-free DNA-stabilization solution, and a PCR blank, i.e., PCR-grade water as template) for each 46 samples sequenced.

### Sample processing for sequencing at JMF

DNA extraction and library preparation were performed as described previously [16]. Briefly, mock communities were ordered as extracted DNA standards (Zymo Research, cat. no. D6311), and a peat soil sample was extracted using a phenol-chloroform extraction method after mechanical lysis (bead-beating) [17]. The V3-V4 or V4 regions of 16S rRNA genes were amplified (30 cycles) using primers 341F and 785R [15] or 515F and 806R [18], respectively, modified with a linker sequence [16] and barcoded (8 cycles) in a CD or unique dual (UD) setup. The barcoded samples were purified and normalized over a SequalPrep™ Normalization Plate Kit (Invitrogen) using a Biomek^®^ NXP Span-8 pipetting robot (Beckman Coulter), pooled and concentrated on columns (Anlaytik Jena). Sequencing libraries were prepared with the Illumina TruSeq Nano Kit and sequenced in paired-end mode (2 × 300 nt; v3 chemistry) on an Illumina MiSeq following the manufacturer’s instructions. The workflow systematically included four negative controls (PCR blanks, i.e., PCR-grade water as template) for each 90 samples sequenced.

### Data analysis

Raw amplicon data were analyzed using IMNGS (www.imngs.org) [4], a platform that integrates a UPARSE-based, de novo OTU-picking strategy [19]. A sequence identity threshold of 97% was used for clustering sequences. Additional parameters were: barcode mismatch tolerated, 1; no. of nucleotides trimmed at each the 5′- and 3′-end, 5; trim quality score, 3; max. expected errors, 3; min. read length, 0; max. read length, 600. Data were first processed without any filtering of OTUs. These primary outputs were then further processed using the desired filtering cutoffs, i.e., (i) by removing singletons only (OTUs represented by only one sequence across all samples), which is a commonly used strategy [20], or (ii) by removing those OTUs that did not occur at least at a defined relative abundance in at least one sample (e.g., 0.5% was a threshold we had proposed previously below which the variation of OTU-specific relative abundances between replicate samples increased exponentially [21]). Phylogenetic trees of resulting representative OTU sequences were constructed in FastTree [22]. Whenever appropriate, closed-reference picking was performed in QIIME v1.9.1 using default settings [3]. Processed data were further analyzed using Rhea for the generation of diversity and composition readouts [21]. The identity of OTUs (i.e., their match to the reference sequences of species included in the defined communities) was assessed using BLAST [23], considering ≥97% sequence identify, ≥90% coverage, and an *e* value <0.00001 as positive hits. The taxonomy of spurious OTUs was assigned using SILVA [24]. Besides the OTU-based approach, the DADA2 pipeline v1.12.1. was used on data from mock communities and gnotobiotes to generate ASV with the recommended settings for paired-end sequences (adjusted options: maxEE, 3.3; truncQ, 3; maxN, 0; truncLeft, 10; truncRight, 20) [6]. Samples processed at JMF were analyzed using DADA2 v1.14.0 following a previously described workflow [25] with pooling for each run (adjusted options: truncLen, 150 for V4; truncLen, 230 for V3-V4; maxEE, adjusted for each run).

### Large-scale amplicon sequencing studies

All spurious OTUs from the mock communities across ten sequencing runs were collapsed at 97% sequence identity using UCLUST [5] to remove redundancy. Samples in IMNGS (build 1905) [4] with unambiguous origin were grouped into five categories (human, mouse, soil, freshwater, and marine). All pre-calculated OTUs in the selected IMNGS samples were searched against the spurious sequences from each run in parallel and assigned to their best match with identity >97% over 90% of the query length. Results were merged into an occurrence map of all spurious OTUs in each IMNGS sample tested. Due to different primers being used across studies, there is no guarantee of overlap between spurious sequences and those from IMNGS samples. Hence, IMNGS samples with no hit to any of the spurious OTUs were not considered as it was unclear if spurious sequences were indeed absent from these samples or regions simply did not match. The prevalence of each spurious OTU in all sample categories was calculated as the percentage of samples in the given category that were positive at a threshold >0.25% relative abundance. When spurious OTUs occurred in different sample categories, a *Z*-test was used to determine whether sequences could be considered as exclusive to one of these sample categories (*p* < 0.05).

### Statistics

Unless otherwise stated, values in the text are presented as mean ± standard deviation. All statistical tests were performed in R, v3.4.0. *P* values < 0.05 were considered as significant (after adjustment for multiple testing whenever appropriate using the Benjamini–Hochberg method). For microbial community analyses, detailed descriptions of statistical tests applied are provided in the Rhea support information and in the corresponding scripts (https://lagkouvardos.github.io/Rhea). Sequence counts were normalized according to the minimum sum count across the given OTU table prior to calculation of *alpha*-diversity parameters. *Beta*-diversity analyses were based on the calculation of unweighted and generalized UniFrac distances [26, 27].

## Results

### Filtering threshold for handling spurious sequences

**Fig. 2:**
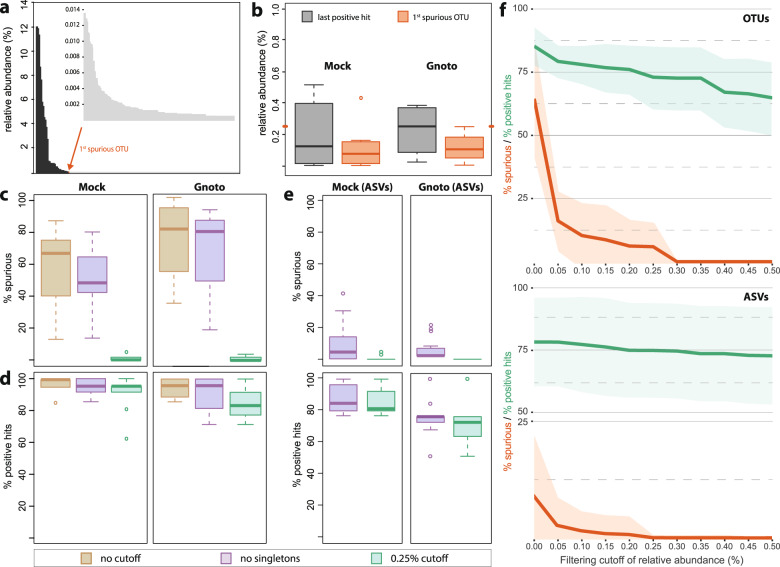
Determination of filtering thresholds using artificial communities of known composition in vitro (mock; *n* = 9 different types; 21 replicates in total) and in mice (gnotobiotes; *n* = 4 different communities; 28 mice in total). **a** Example of the occurrence of all molecular species detected without filtering in the gut of a gnotobiotic mouse [49]. The arrow indicates the position of the first spurious molecular species, all following taxa being considered as having a high risk of being spurious (light gray bars in the enlarged inset). **b** Distribution of the relative abundances of first occurring spurious molecular species (as shown in panel **a**) across all mock communities and samples from gnotobiotes. The orange dashes on the *y*-axis indicate the consensus threshold of 0.25% relative abundance, above which no spurious taxa occurred with the exception of one outlier in a mock community at a relative abundance of 0.44%. **c** Comparison of various standard filtering cutoffs (see explanations in the text) in terms of spurious taxa (i.e., those molecular species not matching sequences of the known species contained in the artificial communities). **d** Corresponding percentages of positive hits retained by the different filtering strategies, with positive hits being defined as the reference sequences found in the respective amplicon datasets. **e** Percentage of spurious taxa and positive hits in the same reference communities using the DADA2 pipeline for analysis based on amplicon sequence variants (ASVs) [6]. **f** Effect of filtering thresholds at increments of 0.05% relative abundance on the detection of spurious taxa and positive hits in all mock and gnotobiotic datasets for OTUs (upper panel) and ASVs (lower panel). Lines correspond to mean values; ribbons represent standard deviations.

We first used bacterial communities of known composition (simplified communities) to assess the occurrence of spurious taxa and to determine at which relative abundances they begin to appear. To propose a cutoff that is potentially applicable to different 16S rRNA gene amplicon studies, we included reference data obtained with different variable regions and sequencing pipelines and originating from both in vitro an in vivo communities varying in number and type of species (max. 58) (Tables 1 and 2). To determine a filtering threshold that allowed exclusion of most spurious taxa, we recorded the relative abundance of the first spurious OTU occurring in each of the reference community datasets (Fig. 2a). Median values of approx. 0.12% relative abundance were observed (Fig. 2b). Besides one outlier in the mock communities (0.44% relative abundance), all values were below 0.25% relative abundance.

Without any filtering, sequence clustering generated an average of 508 ± 355 OTUs (min. 52; max. 1081) per mock community (10–58 target species in theory) and 105 ± 50 OTUs (min, 55; max. 215) per gnotobiotic community (4–12 target species in theory). Up to 87% of these OTUs were spurious (i.e., they did not match the expected classification of species contained in the corresponding artificial community) (Fig. 2c). On average, the proportion of spurious OTUs in both the mock communities and samples from gnotobiotic mice was slightly lower after removing singletons, although this did not reach statistical significance (50.8 vs. 64.3%, *p* = 0.227; 57.5% vs. 65.7%; *p* = 0.70, pairwise comparison by *t*-test, including Benjamini–Hochberg correction following ANOVA). Interestingly, the proportion of spurious molecular species was higher in gnotobiotic mice independent of filtering (*p* < 0.001), suggesting that the matrix accompanying the defined communities (fecal material in this instance) influences the outcome. Besides the goal of removing spurious taxa, it is of course important to include as many true molecular species as possible into the analysis. Even without any cutoff, not all target species could be detected: the percentage of positive hits was 94.9% and 92.3% for mock communities and gnotobiotic mice, respectively (Fig. 2d).

Although the number of spurious taxa decreased drastically (4.0 vs. 50.8% for mock communities and 1.0 vs. 57.0% for gnotobiotes; *p* ≤ 0.01) after applying the proposed cutoff of 0.25% relative abundance vs. singletons removal (Fig. 2c), the number of positive hits was not affected significantly (87.2 vs. 93.7% for mock communities and 82.4 vs. 88.7% for gnotobiotes; *p* > 0.50) (Fig. 2d). Note that the diversity of reference communities in the gnotobiotic mice was relatively low (4–12 members; Table 2), resulting in a marked drop in the percentage of positive hit (8–25%) when even just one true member is excluded after filtering because of its low relative abundance (which is an expectable event considering a classical, exponentially decreasing distribution of species occurrence in gut environments).

We next employed the widely used ASV analysis approach to confirm the aforementioned results. Processing of the same simplified communities generated a total number of 42 ± 25 ASVs (min. 16; max. 98) for mock communities (10–58 target species) and 14 ± 8 ASVs (min. 4; max. 25) for gnotobiotes (4–12 target species). Altogether, a marked decrease in spurious taxa was observed compared with OTU clustering, with an average of 8.6 ± 11.8 and 4.4 ± 6.4% spurious sequences for mock and gnotobiotic communities, respectively (comparison of purple box plots in Fig. 2e, top panels, and Fig. 2c). Of note, the DADA2 pipeline used for the ASV approach does not infer sequence variants that are only supported by a single read (singletons) due to a lack of confidence in their existence relative to sequencing errors. Consequently, data corresponding to “no filtering” with the OTU-based approach were not generated. On average, the first spurious ASV occurred at a relative abundance of 0.10 ± 0.32%. By applying the cutoff of 0.25% relative abundance, spurious sequences were completely removed (except for three outlying samples), albeit with a slight drop in positive hits for both mock and gnotobiotic communities (Fig. 2e).

To obtain a more comprehensive view on how filtering thresholds affect the detection of spurious taxa, all datasets (mock and gnotobiotic mice) were processed using a range of relative abundance filtering thresholds (from 0 to 0.5% at increments of 0.05%) after either OTU- or ASV-based processing of raw sequence reads (Fig. 2f). These data indicate that filtering thresholds between 0.1 and 0.3% are appropriate to reduce the occurrence of spurious taxa to <10% of total OTUs at a loss of <15% positive hits. It is important to note that our intent is not to set a strict rule for data processing, and we recognize that filtering strategies must be adapted in a study-specific manner. Instead, we aim to suggest best-practice guidelines and raise awareness for the importance of proper handling of spurious sequences. To further investigate spurious taxa, the threshold of 0.25% relative abundance described in the first paragraph was kept for all further analyses.

### Ecology and origin of spurious taxa

**Fig. 3:**
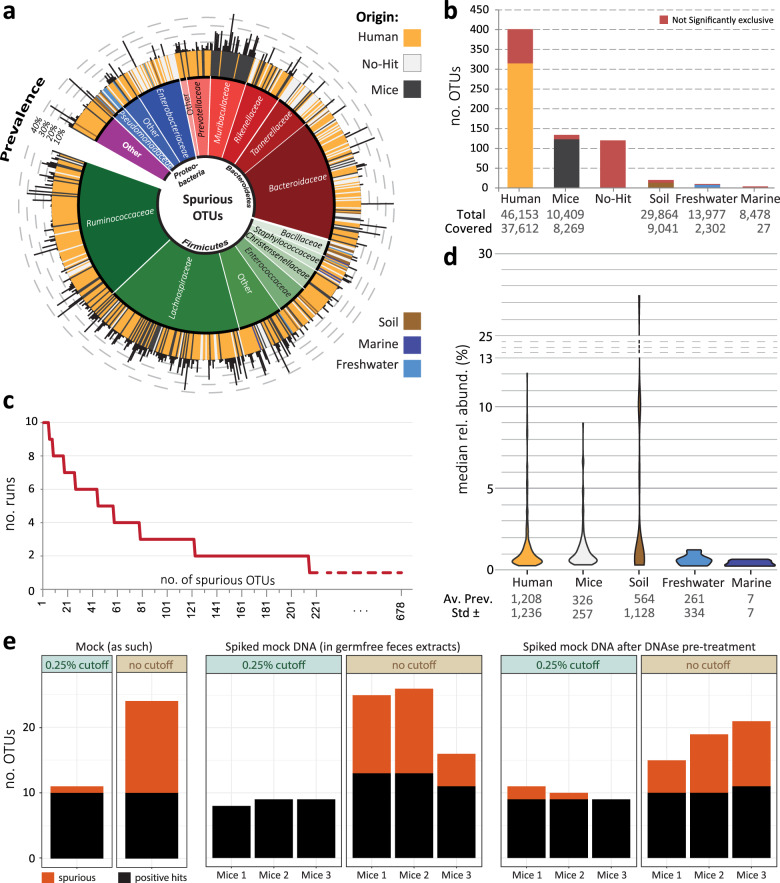
Origin and occurrence of spurious taxa. **a** Taxonomic profile and ecological distribution. Inner ring: SILVA-based classification of all non-redundant spurious molecular species at the phylum and family level. Outer colored ring: sample type characterized by the highest prevalence for the given taxon. Outer bars: corresponding highest prevalence values. Only samples with relative abundances >0.25% for any given OTU were counted as positive for prevalence calculation. The total numbers of samples considered were: human, 46,153; soil, 29,864; freshwater, 13,977; mouse, 10,409; marine, 8478. **b** Distribution of the spurious taxa across sample types. The exclusivity of each OTU for any given sample type was assessed using a *Z*-test: those assumed to be non-specific for any given sample type appear in red (*p* < 0.05). The total number of IMNGS samples considered for each sample type with at least one of any spurious taxa matching sequences above 0.25% relative abundance was labeled as “Total” (equal numbers in panel **a**). The number of samples in each type covered by at least one spurious OTU with highest prevalence in this sample type was labeled as “Covered” (i.e., the remaining samples in that category contained also at least one spurious OTU, which was however characterized by highest prevalence in another sample type). **c** Redundancy of the spurious taxa across 10 sequencing runs. **d** Violin plots of the distribution of median relative abundances of all spurious molecular species within each sample type as shown in panel **b**. The average prevalence of the spurious taxa in each sample category is shown as mean ± SD below the *x*-axis. **e** The ZymoBIOMICS DNA Standard was sequenced as such or in combination with DNA extracts of cecal contents from germfree mice with or without pre-treatment for free DNA removal as described in detail in the methods. The stacked bar plots indicate the number of spurious taxa and positive hits in the different sample treatment categories with or without relative abundance filtering following the color codes presented in the figure panel.

To better understand how spurious molecular species arise in amplicon datasets, we investigated the diversity and origin of sequences not matching reference sequences from the defined communities. To this end, we taxonomically classified and evaluated the occurrence of these sequences in >100,000 IMNGS-derived amplicon data [4]. Approximately half of the 678 non-redundant spurious OTUs belonged to the phylum Firmicutes, followed by Bacteroidetes and Proteobacteria. Most of these were characterized by highest prevalence in human- and mouse-derived datasets, with values reaching up to 40% in the thousands of tested samples (Fig. 3a). Over 20% of spurious molecular species detected in human and mouse samples were only found in these habitats (Fig. 3b). This distribution implies that the type of samples multiplexed with target samples within a given sequencing run (in our case mouse and human gut samples) greatly influences the occurrence of spurious OTUs in target samples. Interestingly, >600 of the 678 spurious OTUs occurred in fewer than five of the ten sequencing runs tested, with approximately 450 of them occurring in only one run (Fig. 3c). This observation indicates that the majority of spurious taxa are sporadic cross-contaminations rather than generalist artifacts across sequencing runs, suggesting that fully independent technical replicates would improve data quality. Although most of the spurious taxa were characterized by relative abundances between 0.25 and 2% in the IMNGS-amplicon datasets tested, they represented very dominant populations in a few samples (Fig. 3d).

To test sample matrix effects and the effect of free DNA removal commonly used for low biomass samples on the occurrence of spurious taxa, mock community DNA was used in combination with gut samples from germfree mice that were either pre-treated for free DNA removal or not (Fig. 1b). While all negative controls (DNA isolation and PCR blanks, treated and untreated control germfree samples) generated <100 processed reads, the average sequencing depth was 14,129 ± 4,682 for the target samples. The digestion of free DNA prior to extraction from germfree cecal contents tended to lower the number of spurious taxa detected; however, these spurious taxa still represented at least one third of all OTUs among the three samples tested without relative abundance filtering (Fig. 3e). Using the 0.25% cutoff provided the most congruent results with respect to the expected taxa diversity in the mock community. However, a few spurious taxa were still present when free DNA removal was employed, and the number of positive hits tended to be higher than the expected number of 8 due to the present of satellite OTUs of low abundance (Fig. 3e).

### Inadequate taxa filtering inflates *alpha*-diversity and increases heterogeneity

Spurious taxa, such as those considered in the present study, tend to be low abundant per se: the cumulative relative abundance of spurious molecular species in the reference communities used above was approx. 1% on average. Consequently, spurious sequences are not expected to substantially influence overall composition data, even though the risk exists that authors draw attention onto spurious taxa characterized by statistical significant, yet biologically irrelevant different. In contrast, spurious sequences can have a major influence on diversity (e.g., richness and evenness for *alpha*-diversity and between-sample distances for *beta*-diversity), as presented in the next sections. To assess the effect of filtering thresholds on analysis outcomes, we used recently published amplicon data from two comprehensive studies that included a substantial number of samples analyzed by Illumina sequencing of 16S rRNA gene amplicons and for which raw datasets could be retrieved from public repositories. The study by Flores et al. [12] (hereon referred to as Study-1) focused on dynamics of human body microbiomes over time, collecting samples weekly from 85 college-age adults over a 3-month period (in the present work, we focused only on the gut samples). The second study published by Halfvarson et al. [11] (hereon referred to as Study-2) focused on shifts in the human fecal microbiota over time in patients with inflammatory bowel diseases vs. controls and consisted of 683 fecal samples from 137 individuals. We emphasize again that the purpose of the present study was not to confirm or refute data from the literature, but rather to draw attention to an analysis parameter that can profoundly affect results. In all the following analyses, outcomes after the common approach of filtering singletons after de novo OTU clustering were compared with the 0.25% cutoff introduced above (i.e., keeping only those OTUs occurring at a minimum relative abundance of 0.25% in at least one sample).

**Fig. 4:**
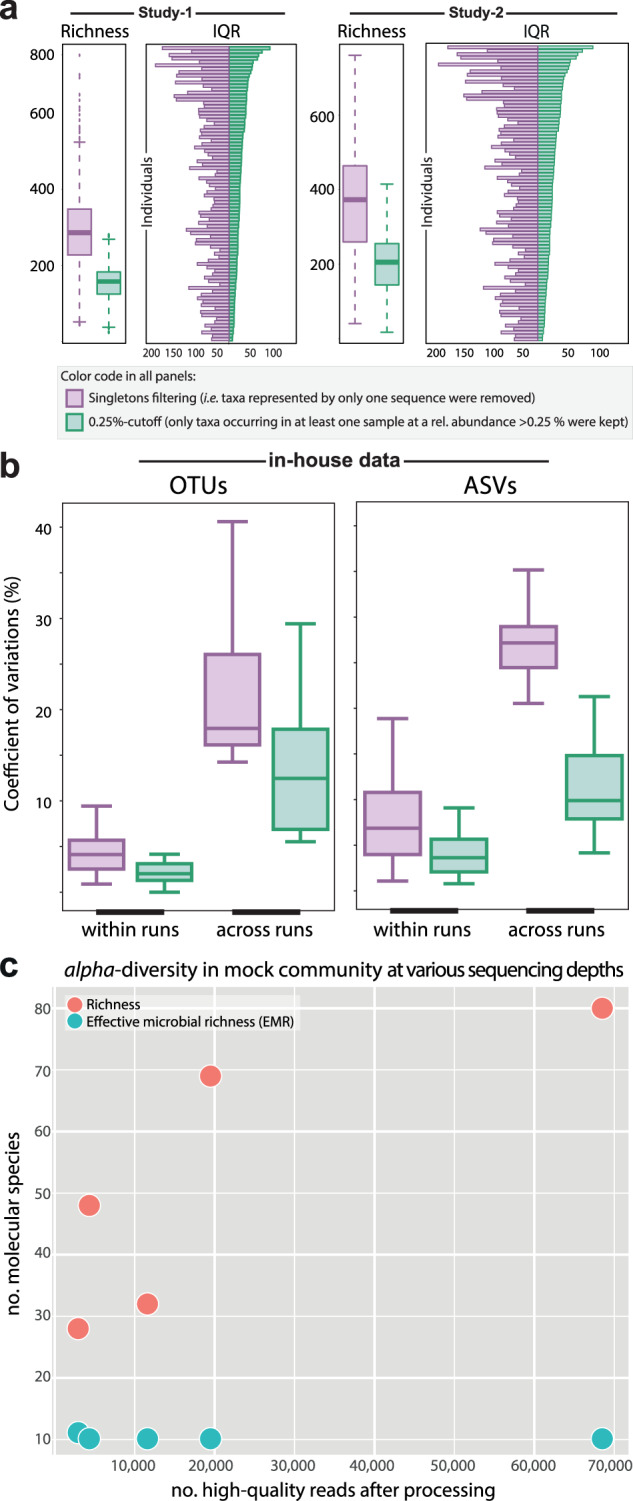
Influence of sequence filtering methods on *alpha*-diversity. **a** Richness distribution across all individual samples and time points. The bar plots show interquartile ranges (IQR = Q3–Q1) of individual samples (rows) as a proxy for richness variation across the various time points of a given sample. IQRs were ranked by decreasing values after applying the 0.25% cutoff. Colors are: purple, singleton removal; green, 0.25% cutoff filtering (i.e., keeping only those molecular species occurring in at least one sample at a relative abundance >0.25%). **b** Coefficient of variations calculated on richness values obtained from six fecal samples each sequenced in triplicates in seven different sequencing runs. Sequencing reads were processed using either an OTU- or ASV-based approach (left or right box, respectively). Within runs: variations across triplicates within any given sequencing run. Across runs: variations between the same samples included in the different runs. **c** Richness and effective microbial richness (see definition in the text) in the ZymoBIOMICS DNA Standard at increasing sequencing depths (*x*-axis).

In both Study-1 and Study-2, filtering OTUs using the 0.25% cutoff led to an approximately two-fold decrease in richness, resulting in an average number of about 200 observed species per sample (Fig. 4a). Interestingly, when looking at individual variations in richness by plotting interquartile ranges (IQR) across the different time points analyzed in the studies, the 0.25% cutoff was associated with a significantly lower heterogeneity in richness (Study-1: IQR = 28.0 ± 17.8 vs. 70.6 ± 34.1, *p* < 0.001; Study-2: IQR = 17.0 ± 3.2 vs. 49.0 ± 10.4, *p* = 2.5 × 10^−13^) (Fig. 4a). Another helpful readout of *alpha*-diversity is the Shannon effective count, which accounts for the evenness of species distribution and can be, simply speaking, considered as a proxy for the number of most dominant species [21, 28]. Altogether, the trend observed for richness (less heterogeneity after 0.25% filtering) was similar when considering Shannon effective counts (data not shown). However, lower effective counts after stringent filtering (0.25%) were not significantly different for Study-2, showing that Shannon effective counts can be useful to alleviate the influence of lowly abundant species.

In addition to these two published studies, which focused on the analysis of different biological samples (i.e., from multiple individuals at several time points), we also analyzed triplicates of six fecal samples from healthy human adults sequenced several times in-house. This dataset, which consisted of the same samples sequenced in seven different runs, allowed us to evaluate technical reproducibility depending on filtering thresholds (Fig. 1c). Across all runs, the coefficient of variations (CVs) calculated on richness values among the triplicates of each sample within a run were on average <5% and lowest when applying the 0.25% cutoff (Fig. 4b). In contrast, CVs of the richness within samples across sequencing runs increased to 20% on average with a peak at 40% when applying singletons filtering, which dropped to approx. 10% (average) and 30% (maximum) when applying the 0.25% cutoff (Wilcoxon Mann–Whitney test, *p* = 0.004) (Fig. 4b). Similar results were obtained when using an ASV-based data processing strategy (Fig. 4b). These data clearly indicate that 16S rRNA gene amplicon sequencing, at least as performed in our study, generates richness values that vary markedly between sequencing runs for the same sample, especially when following a loose taxa filtering strategy such as singleton removal.

To assess the effect of sequencing depth on *alpha*-diversity, we sequenced the ZymoBIOMICS DNA Standard by multiplexing uniquely barcoded amplicon libraries of this reference mock community at different cluster densities. Analysis of all molecular species created after processing without filtering clearly showed that richness inflated as a function of sequencing depth (Fig. 4c). In contrast, the count of taxa occurring above 0.25% relative abundance, referred to as “effective microbial richness” (EMR), was stable and a better proxy of the true diversity within the reference community.

### Between-sample comparisons are influenced by filtering strategies

We next assessed how filtering influenced *beta*-diversity analyses. As in the published studies selected [11, 12], a closed-reference OTU protocol was also used to obtain reference data to which filtering strategies after de novo OTU clustering could be compared.

**Fig. 5:**
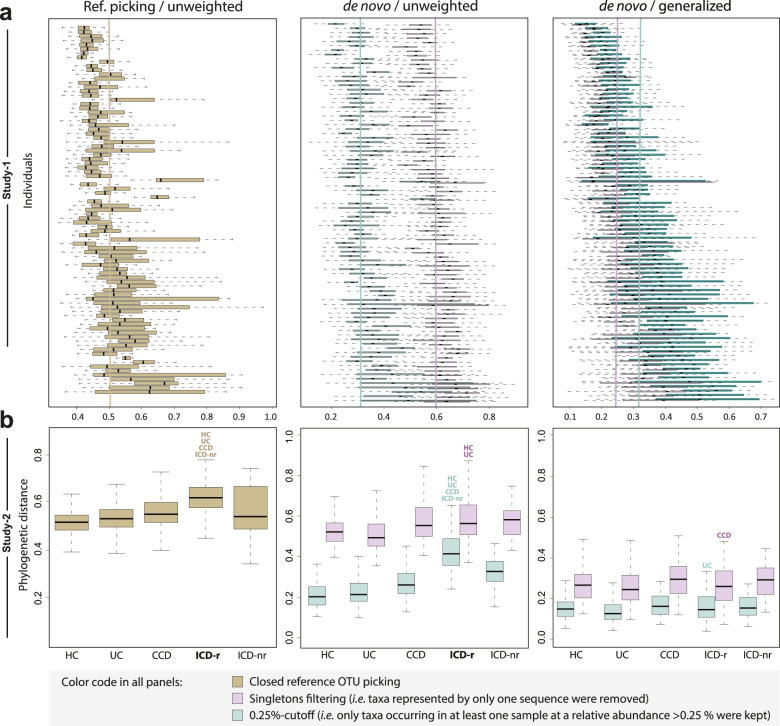
Effect of sequence filtering methods on *beta*-diversity outcomes from literature data. Colors are as in Fig. 4. Brown indicates closed-reference picking. **a** Overtime variations in microbiota profiles for each individual from Study-1 [12] based on reference OTU-picking and unweighted UniFrac distances (left; as in the published study), de novo OTU-picking and unweighted UniFrac distances (middle) or generalized UniFrac distances (right). Bars indicate median distances across all individuals. Individuals were ordered by increasing average distance using the 0.25% cutoff and generalized UniFrac (right panel). **b** Differences in the phylogenetic makeup of fecal microbiota as in panel a for Study-2 [11].

In Study-1, the median unweighted distance across all individuals was approximately 0.5 after using reference-picking, including a broad range of within-host temporal variations (e.g., some individuals were characterized by more stable profiles than others) (Fig. 5a; left panel), as observed in the original study [12]. As expected, the strongest effect of filtering strategies was observed when using unweighted UniFrac distances: singleton removal was characterized by a higher temporal variation in profiles (median value of approx. 0.6 vs. 0.3 for the 0.25% cutoff) (Fig. 5a; middle panel). Notably, using generalized UniFrac distances decreased the difference between filtering approaches; however, it also widened the range of individual-specific temporal variability around the median, potentially enhancing the discriminatory power between “stable” and “variable” individuals (Fig. 5a; right panel).

In Study-2, one of the main findings in the original work was that volatility (i.e., variations overtime within individuals) was highest in patients suffering from Crohn’s disease with an ileal phenotype who underwent ileocecal resection (ICD-r) [11]. We confirmed this finding by using reference-based picking and unweighted distances, as performed in the published manuscript (Fig. 5b; left panel). However, when applying de novo clustering, this difference could only be observed when using the 0.25% cutoff in combination with unweighted distances (Fig. 5b; middle panel). The absence of significant differences when using unweighted distances after singletons removal suggests that the biological signal in this study is overwhelmed by the stochastic “noise” introduced by spurious molecular species (Fig. 5b; middle panel). The absence of differences when applying generalized distances (Fig. 5b; right panel) further suggests that individual-specific temporal variations are attributable to the presence/absence of taxa rather than to changes in composition.

### Validation studies

To confirm the utility of the 0.25% cutoff inferred from the aforementioned data generated at the Core Facility Microbiome of the ZIEL Institute for Food & Health (TU Munich, Germany) and at the Institute of Medical Microbiology of the RWTH University Hospital, additional samples were processed and analyzed independently at the Joint Microbiome Facility of the Medical University of Vienna and the University of Vienna (JMF).

**Fig. 6:**
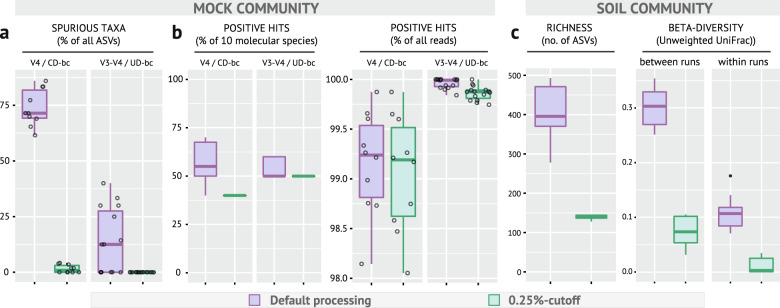
Validation studies in a fully independent sequencing facility. **a** Fraction of spurious taxa with (green) or without (violet) applying the 0.25% cutoff displayed according to the targeted 16S rRNA gene regions and barcoding strategy used. **b** Corresponding fraction of positive hits (i.e., amplicons matching the reference strains contained in the mock community). **c** Average and distribution of richness and distance values between replicates of the same soil sample processed in multiple sequencing runs. ASV amplicon sequence variant, bc barcoding, CD combinatorial dual, no. number, UD unique dual.

First, processing of a log-distributed version of the ZymoBIOMICS Microbial Community Standard (Zymo Research GmbH) containing eight bacterial strains confirmed the advantage of applying the 0.25% filtering approach. Twenty-five replicates of the same DNA sample were sequenced on five sequencing runs (1–8 replicates per run) using either the V4 region combined with CD barcoding or the V3-V4 regions with UD barcoding (two and three runs, respectively). V4/CD vs. V3-V4/UD yielded 31 ± 16 vs. 8 ± 2 ASVs (min: 13 vs. 5, max: 57 vs. 10), respectively. Spurious ASVs (i.e., all sequences with a Hamming distance to the reference >1) were greatly reduced using a 0.25% filtering step, from 73 ± 8 to 2 ± 2 and from 13 ± 15 to 0 in V4/CD vs. V3-V4/UD, respectively (Fig. 6a). This occurred at a loss of 15% true taxa in the case of V4/CD while no change was observed with V3-V4/UD (Fig. 6b). As the highest relative abundance reached by any spurious ASV was 0.28% and the true taxa detected corresponded to dominant members of the standard community, the cumulative relative abundance of true taxa was high (>98%) in all cases (Fig. 6b).

Second, peat soil DNA [17] was analyzed to confirm suitability of our filtering approach for non-gut samples. One identical DNA sample was sequenced on three different runs (3–5 replicates per run) using primers 515F/806R (V4 region) and CD barcoding. The ASV table was rarefied to the minimum sum count (9104) and analyzed with or without filtering (i.e., only ASVs observed at a relative abundance >0.25% in at least one replicate were kept). Richness was calculated using ampvis2 [29]. Applying the 0.25% cutoff decreased the number of observed ASVs from 408 ± 71 to 139 ± 5 and, more importantly, the IQR from 101 to 7 (Fig. 6b). Unweighted UniFrac distances within and between runs as calculated using ampvis2 were also compared before and after filtering. Sequences were aligned using MAFFT [30] and phylogeny was inferred using FastTree. Whilst the community makeup in the soil sample varied substantially between sequencing runs without additional filtering, the 0.25% cutoff reduced this variation to the level observed within runs without filtering (Fig. 6c). Replicates within a run were very similar after applying the 0.25% cutoff. Altogether, these data serve as an independent confirmation that stringent filtering delivers more stable values obtained for the exact same sample sequenced in replicates across several sequencing runs.

## Discussion

The goal of our work was to investigate the occurrence of spurious taxa in high-throughput 16S rRNA gene amplicon datasets. The findings clearly underscore the need for careful treatment and interpretation of lowly abundant sequences.

The advent of high-throughput sequencing has dramatically expanded our understanding of microbial diversity, but has also lead to claims that tens of thousands of species inhabit the human gastrointestinal tract [31] and that sterile organs also have microbiomes [32], a concept that has largely been dismissed due to overwhelming evidence to the contrary [33–35]. Here, we show that filtering sequencing datasets based on the widely used approach of removing singletons is insufficient to exclude a high proportion of spurious taxa. Of course, enhancing the filtering stringency by increasing relative abundance thresholds comes with the risk of losing true diversity. Hence, analysis strategies should always be adapted to the main goal of a study, and no single “optimal” threshold can be defined. It is beyond the scope of the present work to dissect the contribution of each wet lab and in silico step to the introduction of spurious taxa into datasets. Nonetheless, we observed that many spurious taxa most likely originate from samples multiplexed with the defined communities in one sequencing run, despite the implementation of multiple negative controls and an automated sample processing workflow. One cause for spurious sequences, termed index-hopping, was previously identified to account for 0.47% of reads, with samples with the fewest reads being affected the most [36]. As defined in the present study by using defined communities as references, spurious taxa do not necessarily represent true artifacts (i.e., sequences not corresponding to real microbes). Remnant DNA in laboratory materials and reagents [37] or in the feed used for laboratory animals (including germfree models) do originate from existing microbes and may give rise to amplification products that can confound results (especially when the number of PCR cycles are ≥30, as is often used).

There is an obvious need for harmonizing sequencing-based microbiome studies [38, 39]. Although several research groups have examined the effect of sequence quality filtering and sequencing depth [40–44], the influence of lowly abundant, potentially spurious taxa on readouts has been studied in less detail. Variations between replicate samples after Illumina-based amplicon sequencing can be quite high even after singleton removal [45], and the present study stresses the importance of benchmarking platforms using reference communities. Filtering strategies for removal of spurious taxa primarily affect diversity readouts, especially richness, implying that variations in richness that have very often been associated with disturbed microbial ecosystems under disease conditions should be interpreted with care [46, 47]. Richness estimates are strongly dependent on parameters set during bioinformatic analysis. Due to the influence of sequencing depth on measured richness, it is usually normalized for comparison across samples, typically by using the minimum depth across all samples in the study. However, this approach does not help when making comparisons between studies, for which a standardized normalization would be useful. Technically, all taxa must be counted for the estimation of richness, but the existence of spurious taxa in sequencing data requires the implementation of appropriate cutoffs. Legacy has favored the use of singleton removal before estimating richness. However, a singleton from a sample with 100 reads should obviously not be weighted the same as a singleton from another sample with 100,000 reads. That is why proportional filtering thresholds have been applied, albeit with marked variations between studies and with little to no justification. We found that the majority of spurious molecular species can be effectively removed by applying a 0.25% relative abundance cutoff. Although by no means universal, we recommend its usage over singleton removal prior to *alpha*- and *beta*-diversity analyses. Such filtering is simple to implement and already available in IMNGS (www.imngs.org).

Although study-specific filtering is effective in reducing the number of spurious taxa and their effect on diversity measures, its outcome depends on the number of samples included in the study (as any molecular species occurring at a relative abundance above the selected threshold in at least one sample is kept) and the depth of sequencing per sample. Due to this, *alpha*-diversity measures, such as richness, are especially sensitive to the normalization and filtering applied, thus making it difficult to compare richness across studies. A sample-specific measurement of *alpha*-diversity that takes into account the effect of sequencing depth and spurious taxa would be very useful for comparative analysis between studies. We therefore propose the concept of “EMR,” which is defined as the number of taxa with a relative abundance greater than a set cutoff (per default 0.25%) in each microbial profile considered. In other words, EMR is equivalent to the count of taxa after normalization to 1000 reads and removal of those occurring below 2.5 counts. Importantly, EMR is unaffected by sequencing depth or normalization steps (Fig. 4c). Together with other established *alpha*-diversity measures such as Shannon effective counts, EMR is now implemented in Rhea (https://lagkouvardos.github.io/Rhea) to facilitate robust inter-study comparisons.

## Conclusions

Despite the development of new sequencing approaches for studying microbiomes such as shallow metagenomics [48], 16S rRNA gene amplicon sequencing is still being used very widely. Thresholds for filtering lowly abundant taxa in such datasets can markedly influence the outcome of microbiota analysis, especially diversity readouts. We strongly recommend applying filtering strategies that go beyond singleton removal. Applying a minimum relative abundance threshold between 0.10 and 0.30% is superior to singleton removal, although study-specific analysis strategies may be needed depending on, for instance, the type of samples analyzed and the sequencing depth achieved. “EMR” will help facilitate the comparison of *alpha*-diversity across studies.

## Data Availability

The 16S rRNA gene amplicon datasets generated in the present study are available in the European Nucleotide Archive (www.ebi.ac.uk/ena) under study accession number PRJEB34431 (data from the Core Facility Microbiome of ZIEL) and SRA accession numbers SRR10688001-37 (data from the JMF) and PRJNA659641 (data from RWTH Aachen). All scripts and codes used to generate the data in this manuscript can be obtained at 10.5281/zenodo.4837436.
